# Seasonal variations of dissolved organic carbon in precipitation over urban and forest sites in central Poland

**DOI:** 10.1007/s11356-015-4356-3

**Published:** 2015-03-22

**Authors:** Patrycja Siudek, Marcin Frankowski, Jerzy Siepak

**Affiliations:** Department of Water and Soil Analysis, Faculty of Chemistry, Adam Mickiewicz University in Poznań, Umultowska 89b Street, 61-614 Poznań, Poland

**Keywords:** Dissolved organic carbon (DOC), Atmospheric pollution, Atmospheric transport

## Abstract

Spatial and temporal variability of carbon species in rainwater (bulk deposition) was studied for the first time at two sites located in urban area of Poznań City and protected woodland area (Jeziory), in central Poland, between April and December 2013. The mean concentration of total carbon (TC) for the first site was 5.86 mg L^−1^, whereas for the second, 5.21 mg L^−1^. Dissolved organic carbon (DOC) concentration accounted for, on average, 87 and 91 % of total carbon in precipitation at urban and non-urban sites, respectively. Significant changes in TC concentrations in rainwater were observed at both sites, indicating that atmospheric transformation, transport, and removal mechanisms of carbonaceous particles were affected by seasonal fluctuations in biogenic/anthropogenic emission and meteorological conditions (i.e., precipitation height and type, atmospheric transport). During the warm season, the DOC concentration in rainwater was mostly influenced by mixed natural and anthropogenic sources. In contrast, during the cold season, the DOC concentration significantly increased mainly as a result of anthropogenic activities, i.e., intensive coal combustion, domestic wood burning, high-temperature processes, etc. In addition, during the winter measurements, significant differences in mean DOC concentration (Kruskal-Wallis test, *p* < 0.05) were determined for rain, mixed rain-snow, and snow samples. It was found that rainwater TOC concentration measured in Poznań and Jeziory reflected a combination of local, regional, and distant sources. Backward trajectory analysis showed that air masses advected from polluted regions in western Europe largely affect the DOC amount in rainwater, both at urban and non-urban sites. These data imply that carbonaceous compounds are of crucial importance in atmospheric chemistry and should be considered as an important parameter while considering wet deposition, reactions with different substances, especially over polluted environments.

## Introduction

Light-absorbing carbon aggregates in the atmosphere affect cloud formation and photochemical transformations (Ramanathan and Carmichael 2008). Previous studies showed that organic carbon can contribute to precipitation acidity and complexation effect (Avery et al. 2013; Mead et al. 2013; Witt et al. 2007). Atmospheric organic carbon exists mainly in gaseous and aerosol phase and can be associated with both anthropogenic (urban/industrial emission) and biogenic (vegetation, large-scale biomass burning of forests, sugar cane foliage and bagasse) sources. It was calculated that annual global rainwater dissolved organic carbon (DOC) flux may equal 0.43 Gt C year^−1^; however, over urban environments, the concentration and deposition fluxes of organic carbon are most often elevated as compared to rural areas (Willey et al. 2000). Based on previous experiments, it was showed that approximately 20–30 % of dissolved organic carbon present in atmospheric water can be attributed to combustion processes (Avery et al. 2006). In particular, during the wintertime, elemental (EC) and organic carbon (OC) in the atmosphere can substantially increase as a result of intensive coal burning (brown carbon). Other sources of atmospheric carbon such as petrochemical industry (fertilizers, catalyst regeneration facilities), traffic (road dust and vehicle emission), and smelters are typical of urban areas. Zhang et al. (2007) stated that aerosol particles in fine mode might contain, on average, half of organic species which could be incorporated into hydrometeors. Despite previous observations focused on the characteristics of carbonaceous aerosol, there are still not enough data on DOC measurements in rainwater from the central and eastern Europe.

In the present study, the main objectives were to investigate carbon compounds in precipitation, collected simultaneously at urban and forest areas, and to find the contribution of various biogenic and anthropogenic emission sources. This preliminary, 10-month carbon project included the analysis of 66 rainwater samples collected in all seasons to provide the full characteristics of total carbon variability over the urbanized region. To our knowledge, this is the first insight into the spatiotemporal distribution of carbon compounds in precipitation over Poland. Poznań is a medium-sized city in Wielkopolska Province (central Poland) with the area of 261.8 km^2^ and population density up to 600 000, characterized by high industrial, urban, and traffic emission. It is a well-situated receptor site to investigate local and regional emission of air pollution as well as to examine aerosol properties and long-range transport of carbonaceous particles originated from the surrounding countries, e.g., Germany, Belarus, Ukraine, Lithuania, Russia, Czech Republic, Slovakia, and Scandinavian countries. Finally, based on the results from other worldwide observations, the effect of geographical and temporal variability of organic compounds in atmospheric water was characterized in the present paper.

## Materials and methods

### Study area

**Fig. 1:**
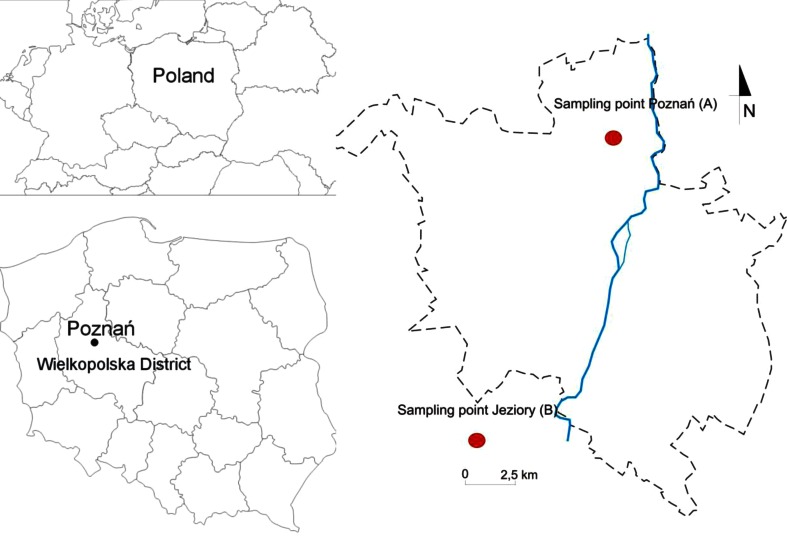
Map of the sampling sites in Poznań (**a**) and Jeziory (**b**), central Poland

Rainwater samples for carbon form, i.e., total carbon (TC), inorganic carbon (IC), and total organic carbon (TOC), analysis were collected simultaneously at two sites in central Poland between April and December 2013. The first sampling site (A) was located in an industrialized and urbanized region of Poznań City, in the Botanic Garden of Adam Mickiewicz University (52° 42′ N, 16° 88′ E, Fig. 1). The AMU garden is situated in the northwest part of the urban area. An international airport Poznań Ławica is located ca. 4 km west of the site. About 10 km northeast of the sampling area, there is a large power plant—Karolin CFPP. Other important air pollution sources close to the first observation site are as follows: dumping grounds for municipal wastes, hospital and domestic sewage, cement factories, sewage treatment plants, industrial units producing metal and paints, smelters, waste incinerators, different manufactories, heavy traffic, and agriculture activities resulting in primary emissions from biomass burning. In addition, the area of the sampling site has significant traffic flow rate. Recent reports of Air Pollution Agency (2013) showed high level of pollution in this region, the annual PM10 concentration in Poznań was 24.8 μg m^−3^.

The second measurement site (B) was situated at the Ecological Station of Adam Mickiewicz University in Jeziory (Fig. 1). This station is located about 30 km southwest of Poznań Agglomeration, in the protected woodland area of Wielkopolski National Park. As for the topography, the Jeziory station is located in morainic plateau at the height of about 130 m above the mean sea level. There is a lake (100 ha) 50 m from the site, and the area is surrounded by a mixed pine-oak forest. There are no particular local anthropogenic urban/industrial activities within this region, and traffic emission is relatively low (medium-traffic road is ca. 4 km away). Hence, the second sampling station can be regarded as a regional background site for atmospheric pollution measurements. Detailed description of this location can be found in the paper by Walna and Kurzyca (2007).

### Sample collection, handling, and analysis

At both locations, different types of precipitation (rain, snow, and mixed) were collected on event basis, using a manually operated bulk precipitation sampler, placed at 1.5 m above ground level. The sampling system consisted of a polyethylene funnel (36 cm diameter) directly connected by a Teflon adaptor to a 1-L acid-cleaned HDPE bottle. Bottles used for rainwater collection were rigorously prepared according to the standard cleaning procedure: rinsing with double deionized water (DDW), soaking with 10 % nitric acid for 48 h, rinsing with DDW, and drying and storing in zip-locked bags. After each rain episode, the sampling set was cleaned with DDW, and the plastic bottle was replaced with a new one, while the collected samples were transported to the laboratory and then stored at 4 °C until the main analysis. Snowfall samples were melted thoroughly at room temperature. The precipitation amount (in mm) was measured manually using a Hellman rain gauge. During the study period, rain events in Poznań ranged from 0.2 to 47.1 mm, whereas in Jeziory, they were between 0.7 and 24.7 mm, with the mean values of 4.3 and 6.9 mm, respectively.

In our study, the samples were filtered through 0.45-μm cellulose nitrate membrane filters (Sartorius) and aliquots were stored at 4 °C in Falcon vials for further analysis (storage time <1 week). Some previous experiments with rainwater kept at 4 °C showed that DOC concentrations were stable (±3 %) for a week (Willey et al. 2000). Other authors also recommended low storage time (ca. 1 week) for filtered rainwater samples or alternatively frozen (ca. 1 year), due to risk of DOC losses caused by microbial activities (Campos et al. 2007; Kieber et al. 2002). A series of initial filtration tests was performed to find any uncertainties during the filtration procedure. The filtration procedure was applied in previous works, especially for the samples with a large amount of particulate organic matter (Willey et al. 2000; Coelho et al. 2008; Pan et al. 2010). Sempére and Kawamura (1996) reported that organic carbon in rainwater is mainly in dissolved fraction (90 %). Following this approach, total carbon was defined as a sum of organic carbon including purgeable organic carbon (POC), dissolved organic fractions (DOC), and inorganic carbon (IC) compounds. In our investigations, the amount of total organic carbon (TOC) measured in filtered rainwater samples was equal to DOC amount.

Concentrations of total carbon (TC), inorganic carbon (IC), and total organic carbon (TOC) in filtered rainwater samples were determined by high-temperature combustion (HTC) using a Shimadzu TOC-L Total Organic Carbon analyzer (Shimadzu, Japan). The solutions for TC determinations were prepared from reagent grade potassium hydrogen phthalate (KHP), while for IC measurements, the mixture of anhydrous sodium carbonate and sodium hydrogen carbonate was used. The analysis of standard solutions and samples ran as follows: 100 μL of a sample solution was injected into a heated catalyst tube to determine TC (combustion temperature 720 °C), a new aliquot was acidified to pH = 2 with H_3_PO_4_ (25 %, *v*/*v*) and purged with CO_2_-free carrier gas (automatic acid addition, carrier gas flow rate = 150 mL min^−1^) to measure the level of CO_2_ produced by inorganic carbon, the sample was subsequently injected into a catalyst tube, and CO_2_ concentration (direct product of carbon combustion) was quantitatively measured by a non-dispersive infrared detector. The instrument was optimized using six-point calibration curve for TC and IC analytical methods. The syringe standard dilution function was used to obtain 1.0, 2.5, and 5.0 mg L^−1^ concentrations from the first (5.0 mg L^−1^) standard solution and 10, 25, and 50 mg L^−1^ from the second (50 mg L^−1^) standard solution. An average percentage recovery of TC was 98.0 ± 2.2 %, and the method precision (given as a variation coefficient) was found at 2.0 % (*n* = 6). The field, procedural, and instrument blanks were analyzed in the same manner as environmental samples and showed no evidence of contamination during the collection, handling, transport, and storage procedure. The detection limit for TC analysis, calculated as three times the standard deviation of a set of six blanks, was 0.04 mg L^−1^.

Additionally, pH and EC values were determined in unfiltered samples through the use of a portable instrument (multi-parameter SevenGo Duo, Mettler Toledo). The total of 66 rainwater samples were determined by average an pH value of 5.6, and 5 % of data had pH lower than 4.4. The range of EC measurements was 5.38–110.7 μS cm^−1^ (on average: 36.8 ± 22.3 μS cm^−1^), whereas the vast majority of snow samples (90 %) had EC values between 11.1 and 77.8 μS cm^−1^.

### Chemical and meteorological data analysis

The statistical analysis of organic carbon concentration for each precipitation event was performed using Statistica 10.0 software. Data were checked for normality and outliers, and distribution pattern was performed to examine differences between sampling locations and seasonal variability. It should be also mentioned that since the considered variables (DOC, TC, and IC concentrations; pH; and EC) were mostly non-normally distributed, non-parametric (i.e., Kruskal-Wallis) tests were applied to determine statistical significance. Meteorological parameters, i.e., air temperature, atmospheric pressure, relative humidity, wind speed, and direction, were registered simultaneously on local weather stations.

In order to characterize air masses passing over both sites, data obtained from the HYSPLIT model were used (NOAA Air Resources Laboratory, Silver Spring, MD, USA, Draxler and Rophl 2003). The input parameters were as follows: meteorological database, GDAS; starting heights, 500/1000/1500 m above ground level; trajectory duration, 72-h; and vertical motion based on model vertical velocity. The 3-day air parcel backward trajectories (BT) with 6-h intervals (at 0:00 am, 6:00 am, 12:00 pm, and 6:00 pm) were generated for each rain episode, separately at Poznań (52.42° N 16.88° E) and Jeziory (52.16° N, 16.48° E) sites. This approach allowed to identify potential sources within the study domain during the long-range transport of air parcels towards the sampling location. The total of 66 trajectories was calculated. Due to the limited range of this paper, we discussed only the most interesting air-mass backward trajectory simulations, which were attributed to rain episodes with elevated concentrations of TOC in Poznań and Jeziory during summertime and wintertime.

During the observation period (April–December), the mean air temperature, pressure, and relative humidity in Poznań ranged between −5.7 and 23.6 ° C, 1000 and 1008 hPa, and 76 and 95 %, respectively. At Jeziory measurement site, air temperature varied between −10.0 and 24.8 °C and relative humidity was, on average, 66 %. The annual precipitation amount in Poznań is 471.4 mm, with the highest monthly value of 98.6 mm in June. Since the impact of coal combustion processes on atmospheric chemistry of carbonaceous species in this area is evident between mid-October and late April (industrial activities related to “heating season”), the special attention was given to relevant months included to study period 2013.

## Results and discussion

### Organic and inorganic carbon in precipitation

**Fig. 2:**
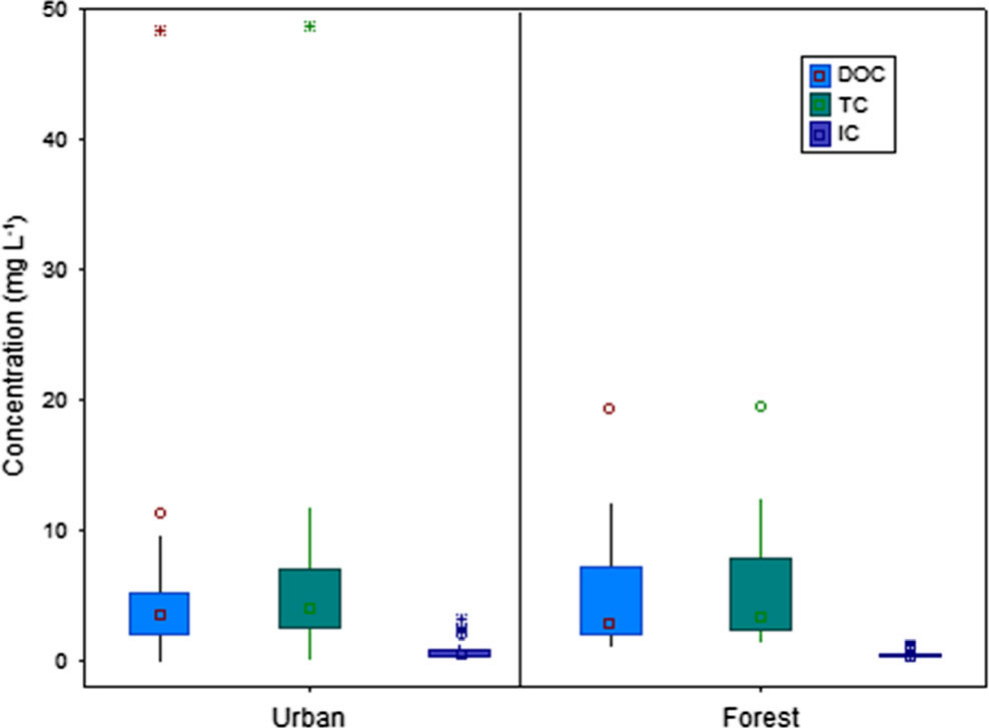
Concentration of DOC, TC, and IC (mg L^−1^) in precipitation over Poznań and Jeziory between April and December 2013 (*boxes* indicate upper and lower quartile, *whiskers* indicate minimum and maximum, *open circles* indicate outlier value, and *asterisks* indicate extreme value)

The concentrations of total organic and inorganic carbon compounds were determined in rainwater samples collected simultaneously at Poznań (*n* = 40) and Jeziory (*n* = 26) sites in central Poland. The Kruskal-Wallis test showed statistically significant differences in rainwater DOC mean concentrations at both locations (*p* = 0.001) during the sampling period in 2013. Overall, the levels of total organic carbon in precipitation at the urban site were slightly higher than those at the protected woodland site (Fig. 2). The average value of rainwater DOC fraction was 5.10 ± 7.46 and 4.72 ± 4.21 mg L^−1^ for Poznań and Jeziory, respectively. The 75 % of DOC measurements obtained for Poznań were characterized by concentration levels up to 5.24 mg L^−1^, whereas the upper quartile for Jeziory corresponded to DOC values <7.20 mg L^−1^ (Fig. 2).

While the concentration of total carbon in precipitation over Poznań ranged from 0.18 to 48.7 mg L^−1^, the values for the second station, in Jeziory, were quite different: 1.59–19.53 mg L^−1^. This suggests higher loading of carbon-related particles in rainwater samples collected at the urban site, associated mostly with the local pollution sources: coal combustion, traffic, construction works, etc. In Poznań, the highest TC level in precipitation was observed in December (pH = 4.4, *H* = 0.4 mm), while rainwater with the lowest TC value was collected in April (pH = 6.53, *H* = 3.6 mm). In contrast to Poznań, the highest TC concentration in rain sampled at Jeziory was found in warm season (August), suggesting biogenic emission as a primary source of organic carbon compounds at this location.

**Fig. 3:**
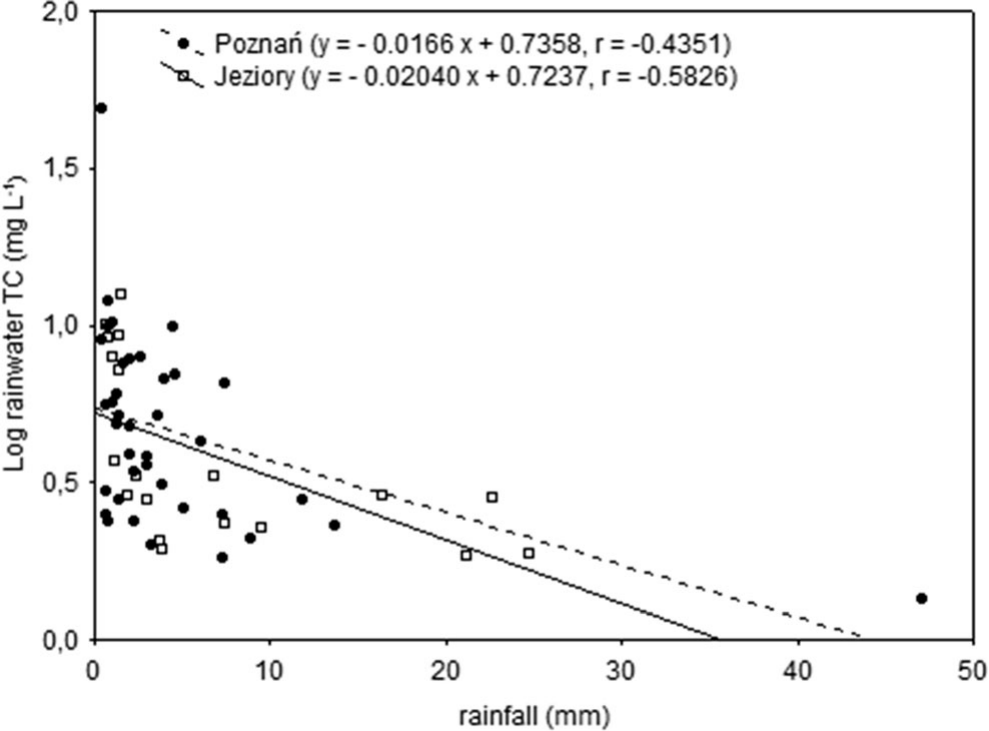
Correlations between precipitation and TC concentration during the sampling period (April–December 2013) in Poznań and Jeziory

In the present study, significant negative linear correlation between precipitation height and total carbon concentration in rainwater was observed for both sites (coefficients of −0.4351 and −0.5826 with 95 % confidence level for Poznań and Jeziory, respectively). It was noticed that regardless of the spatial and seasonal influence, TC concentrations in precipitation increased with decreasing rainfall (Fig. 3). Thus, it can be assumed that the removal (scavenging) of carbonaceous aerosol particles and soluble organic gases from the atmosphere, especially in the initial phase of the precipitation, and the dilution effect have a major impact on TC level in precipitation. In Poznań, due to the larger and continuous contribution of various organic compounds from local urban/industrial sources, the carbonaceous particles were probably not effectively scavenged by first rain. Similar relationships between DOC concentrations and precipitation amount were observed during 1-year study at 10 sites in Northern China (Pan et al. 2010).

### Carbon compounds in precipitation—comparison with other locations

The DOC concentrations measured in rainwater samples at the both sites in Poland and in other locations worldwide are summarized in Table 1. It should be noted that the research on dissolved organic carbon in precipitation has not been previously carried out in Poland. Moreover, to our knowledge, no similar observations were performed for other sites in central and eastern Europe, except for few measurement campaigns in Croatia (Orlović-Leko et al. 2009).

**Table 1:** Comparison of rainwater DOC concentrations at two monitoring stations in central Poland and other studies

Location	Type of site	DOC (mg L^−1^)	Reference
Poznań, Poland	Urban	5.10	This study
Jeziory, Poland	Regional background	4.72	
Seoul, Korea	Urban	0.18–9.36	Yan and Kim 2012
Dunedin, New Zealand	Coastal	0.70	Kieber et al. 2002
		0.12–4.81	
Lower Wisconsin River Valley, Wisconsin, USA	Forest	2.9 ± 0.2	Quideau and Bockheim 1997
Hubbard Brook, New Hampshire, USA	Forest	1.1	McDowell and Likens 1988
Guandaushi, Taiwan	Subtropical forest	4.7 ± 2.9	Liu and Sheu 2003
Araraquara, Brasil	Suburban	4.06	Coelho et al. 2008
10 sites in Northern China	Various	2.4–3.9	Pan et al. 2010

The mean concentration of dissolved organic carbon in rainwater samples from Poznań was higher than values reported for other locations in the USA, Europe, and Asia (Table 1). Such large discrepancies between the compared regions might be partly explained by the various origins of TC in precipitation (traffic, biomass burning aerosols, coal combustion, industrial activities, etc.), meteorological situations, various measurement techniques, and differences in the sampling time. In Poznań, rainwater samples exhibited much higher concentrations of DOC than those collected at marine locations, i.e., El Verde (0.62 mg L^−1^, McDowell et al. 1990) or Costa Rica (0.70 mg L^−1^, Eklund et al. 1997). The mean rainwater DOC concentration in Poznań was also seven times higher than that observed at a coastal site in New Zealand (Kieber et al. 2002). At the suburban site in Brazil, mean rainwater DOC concentrations varied between 2.4 and 3.9 mg L^−1^ and were mainly associated with biomass burning processes (Coelho et al. 2008). In their study, DOC concentrations in rainwater gradually increased to the level >12 mg L^−1^ over the whole harvest/dry period, with one rain episode characterized by extremely high level of DOC (204 mg L^−1^).

As shown in Table 1, rainwater collected at Jeziory had the same mean concentration of dissolved organic carbon as that reported by Liu and Sheu (2003), however, much higher as compared to other sites representing forest ecosystem. For instance, McDowell and Likens (1988) noted that the mean DOC concentration in precipitation collected at hardwood stand in Hubbard Brook did not exceed 1.1 mg L^−1^, whereas Quideau and Bockheim (1997) reported DOC concentration of 2.9 mg L^−1^ for the site represented by conifer stand. In the present study, due to the limited range of measurements, we did not determine TC concentration in throughfall samples; however, earlier investigations by Liu and Sheu (2003) indicated that DOC concentrations in these samples might be twofold higher. This statement is also in agreement with theoretical and empirical data that demonstrate how significantly the concentration of soluble and particulate forms of organic carbon can increase when precipitation passes through the forest canopy, which consequently introduces large amount of carbon to the soil system.

Some measurements carried out in the USA and Asia (Avery et al. 2006, 2013) showed that hydrophobic fraction of DOC in rainwater can be derived both from anthropogenic emission (industrial metallurgical processes, waste incineration, steel industry) and marine/terrestrial biogenic sources. Recent study by Grote et al. (2014) demonstrated the significant role of civil aviation in carbonaceous particles budget, which contributes approximately 2.0–2.5 % to the total anthropogenic emission of CO_2_. This source might have a significant influence on the urban site from the present study due to proximity of the airport Poznań-Ławica. Interestingly, 45 % of all rainwater samples collected at Poznań site were attributed to the western wind sector which revealed a large input potential of carbonaceous compounds (i.e., dust layers, fine aerosols) originated from aircraft emission to the total DOC level measured in precipitation.

In the present study, the DOC contribution to rainwater carbon was predominant, constituting, on average, 87 and 91 % of TC in Poznań and Jeziory, respectively. This is slightly higher as compared to data reported for 10 sites in Northern China by Pan et al. (2010). In addition, they found DOC/TOC ratio of 0.79 that explained high contribution of dissolved carbon species to TOC fraction. Willey et al. (2000) identified similar or slightly lower DOC vs. TOC ratio. In our study, mean concentrations of inorganic carbon in rainwater were roughly similar at the both sites (urban 0.43 mg L^−^ and forest 0.40 mg L^−1^). The same range of IC concentrations was determined in rainwater samples from Piracicaba River Basin, southeast Brazil (Lara et al. 2001). However, the observed contribution of inorganic carbon species was lower than that for urban sites, such as Beijing, Baoding, Cangzhou, and Xinglong in northern China, indicating that rainfalls from polluted regions contain larger fraction of carbonate carbon associated with atmospheric particulate matter (Pan et al. 2010).

### Temporal and spatial variations of DOC in rainwater

Some previous investigations reported strong seasonal variations of organic carbon in rainwater (Orlović-Leko et al. 2009; Pan et al. 2010). Results of these studies indicated that the concentration and wet deposition of carbonaceous particles can be explained by a combination of meteorological conditions (i.e., ambient temperature, relative humidity, precipitation amount), site characteristics, and proximity to the major anthropogenic/biogenic emission sources.

**Fig. 4:**
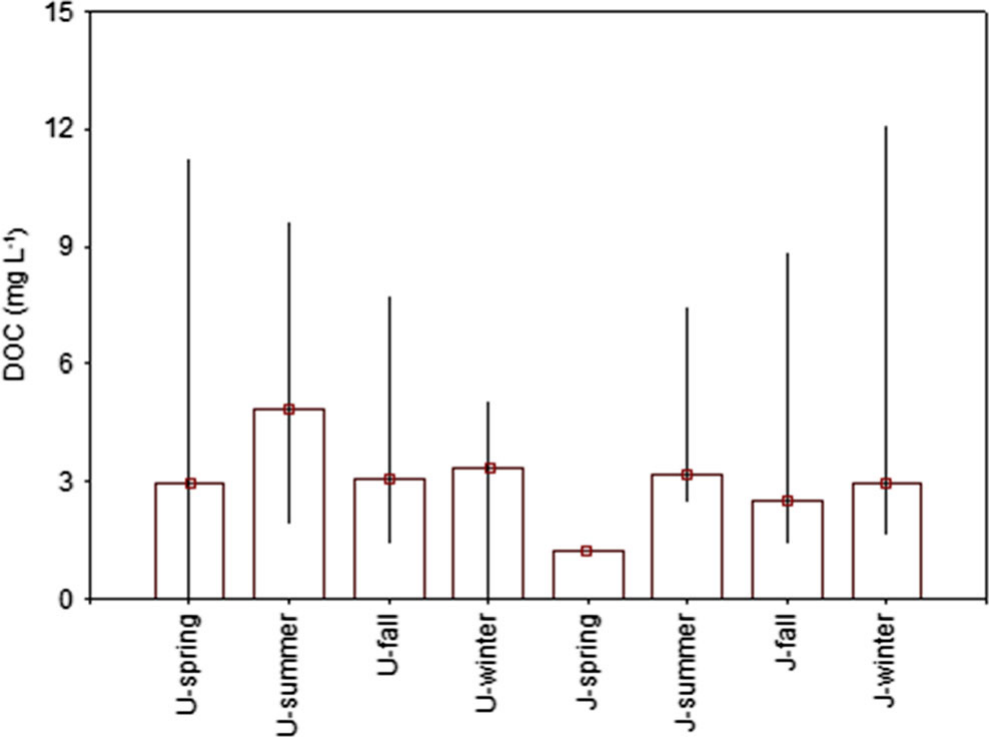
Comparison of DOC concentrations (mg L^−1^) in precipitation obtained for urban (*U*) and forest (*J*) sites during measurement campaigns (spring, summer, fall, and winter) in 2013. The outlier and extreme values of rainwater DOC for Poznań and Jeziory were excluded from the analysis

Figure 4 shows seasonal pattern of DOC in precipitation collected at both stations in the following seasons: spring (April to June), summer (July to August), fall (September to October), and winter (November and December). Overall, DOC concentrations in rainwater had slightly different seasonal distributions, suggesting large impact of different sources on organic carbon reservoir and its transformation in the atmosphere. At Jeziory, the highest DOC level (19.3 mg L^−1^) was observed in summer, whereas the maxima of DOC in rainwater collected in Poznań were found during winter measurements (48.3 mg L^−1^). These results are consistent with data reported by Genberg et al. (2011) during 1-year measurements at Vavihill background station in southern Sweden, indicating that an intensive anthropogenic emission can largely contribute to the increase in carbonaceous particle emission and affect their atmospheric transformations.

The average value of DOC concentration in rainwater sampled during the summer months at Jeziory site was approximately twice as high as in spring (Fig. 4). The summer peak concentration of DOC in precipitation at this site was quite similar to the results obtained by Kieber et al. (2002) and Willey et al. (2000). In contrast, Yan and Kim (2012) during measurement campaigns at the marine site in Seoul (Korea) reported slightly lower values of DOC concentration in rainwater samples between April and September (warm period) as compared to the cold period (October–March). For other locations representing forest ecosystem, the contribution of atmospheric carbon species originated from vegetation (biogenic emission) typically increased during summer period (midst of growing season). High DOC concentrations measured in precipitation during summer months at Jeziory site suggested that local biogenic emission from vegetation was probably the major source of different organic compounds at this area. It is consistent with data from the earlier study by Orlović-Leko et al. (2009) carried out in Šibenik, Croatia. Their results showed mean rainwater DOC concentrations higher by a factor of 1.78 during the warm season compared to non-growing sampling period. Similar trends were also observed by Liu and Sheu (2003) in a single subtropical forest area in Guandaushi (Taiwan), where high level of dissolved organic carbon in precipitation was attributed to significant contribution of low molecular mass organics from local biogenic emission.

In the present study, depending on the site, the gradual decrease of dissolved organic carbon in precipitation by 10–30 % was found between summer and fall. As shown in Fig. 4, rainwater DOC concentrations during September and October (fall) varied between 1.48 and 8.89 mg L^−1^, with the mean value of 4.06 mg L^−1^. It was slightly lower than during winter months, when the mean DOC concentration increased to 12.1 mg L^−1^. The wintertime enhancement in DOC at the urban site can be explained by the presence of carbonaceous aerosol emitted from nearby residential combustion sources or by the transport from Poznań Agglomeration.

It is well known that coal combustion is the largest source of various pollutants over many industrialized and urbanized areas. The greatest emission from this source occurs during winter period, affecting large inter-seasonal variability of carbonaceous aerosol in the local atmosphere. The observed distribution of DOC concentrations in precipitation at our urban site exhibited high variation during the cold season, with the mean value of 3.45 mg L^−1^ in fall and 9.80 mg L^−1^ in winter. In Poznań, the elevated concentrations of dissolved organic carbon in precipitation collected during the wintertime were largely attributed to anthropogenic processes. Recent source apportionment analysis showed that important sources of air pollution in this area primarily include anthropogenic emission from residential coal and wood combustion, industrial and manufacturing plants, traffic (both diesel and gasoline exhausts) and road dust (a study by Siudek, non-published). Previous studies in Sweden demonstrated relatively higher contribution of carbonaceous aerosol related to fossil fuel combustion during the winter season than in summer. The authors argued that the large portion of organic carbon (OC) in fine aerosol, sampled during the cold season, could be attributed to secondary organic aerosol (SOA), because low temperature alters the gas-particle equilibrium (Genberg et al. 2011). Raymond (2005) showed that ca. 20–30 % of rainwater DOC was derived from fossil fuel combustion. Avery et al. (2006) made a suggestion, based on the results of carbon isotopic analysis, that 0.046 Gt C of incompletely combusted fossil fuel could be directly removed from the atmosphere through precipitation. Moreover, measurements by Yan and Kim (2012) showed that within an urban study domain, fossil fuel combustion sources were represented by a large group of activities such as transportation, coal-/oil-firing electricity generators, house heating, and metallurgical processes. All the above mentioned examples provided important information for our study, suggesting that anthropogenic carbon was the main component of atmospheric particles measured in both locations and during all seasons. However, it should be highlighted that an intensive use of brown coal for house heating between mid-October and late February was the main factor responsible for peak concentrations of total carbon in precipitation in Poznań.

Regional dynamics of cloud processes is another important factor controlling carbon budget in the atmosphere (Ramanathan and Carmichael 2008). The relationship between DOC concentration and precipitation type should be considered in this context. The measurements in the period between October and December 2013 in Poznań included various types of precipitation, i.e., rainfalls (R), snowfalls (S), and mixed precipitation (M). Significant differences in mean DOC concentrations (Kruskal-Wallis test, *p* < 0.05) were observed for them: R 9.25 mg L^−1^ (*n* = 10), S 2.62 mg L^−1^ (*n* = 2), and M 1.39 mg L^−1^ (*n* = 2). These results suggest that rainfall was enriched in organic carbon components to a higher extent than snowflakes or mixed precipitation. Previous measurements by Pan et al. (2010) also reported significant differences in DOC concentrations between rain, snow, and mixed samples; however, higher values were associated with snowfalls. In Poznań, the maximum DOC concentration (48.3 mg L^−1^), determined for the rain episode in December, was the result of high PM_10_ emission (47.8 μg m^−3^) and low precipitation amount (*H* = 0.4 mm). In addition, the values of DOC obtained for other meteorological parameters, i.e., low air temperature (−2.7 °C), low mixing heights, low wind speed from southern directions (1.6 m s−^1^) and relatively stagnant atmospheric conditions, suggested that locally emitted pollutants were preferentially trapped over the study area, leading to elevated concentrations of both PM_10_ and PM_2.5_. It should be noted that due to relatively low number of snowfalls and mixed precipitation events (*n* = 2) registered during the winter campaign, the observed pattern R > S > M may be overestimated. Hence, further investigations in this field should be undertaken in order to verify the given hypothesis and to better understand the removal mechanism for organic carbon in the atmosphere in relation to different types of precipitation. Taking into consideration the characteristics of both sampling sites, the observed seasonal changes are not surprising. As mentioned above, the first site (Poznań) was exposed to various types of anthropogenic emission sources, while the second sampling location in Jeziory was affected by milder pollution conditions due to longer distance to emission sources in Poznań Agglomeration.

### Impact of local, regional, and distant sources on DOC levels on rainfalls from central Poland

**Fig. 5:**
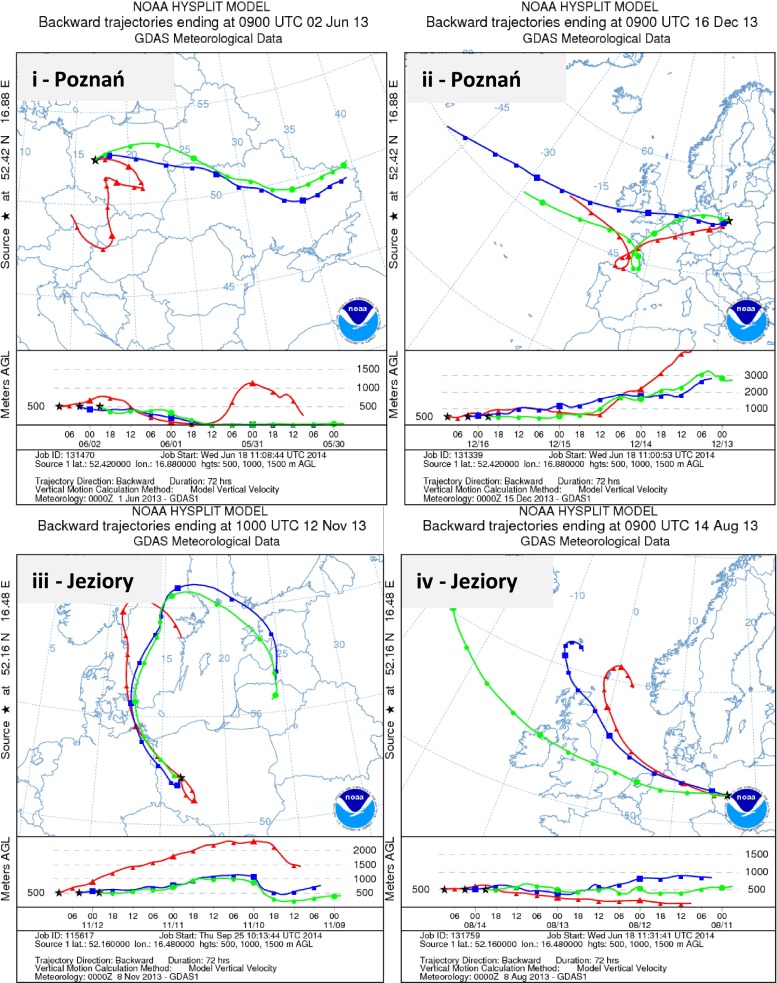
Seventy-two hour back trajectories related to winter and summer conditions, calculated for rain episodes over the urban and forest sites in central Poland. Plots present very high DOC concentrations, including maxima in (*ii*) Poznań on 16th December (48.3 mg L^−1^), (*iv*) Jeziory (19.3 mg L^−1^), and two other cases (*i*, *ii*) when DOC_rain_ > 10 mg L^−1^. Ground levels are marked as follows: 500 m (*red*), 1000 m (*blue*), and 1500 m (*green*)

Recent studies on dissolved organic matter complexity in rainwater conducted by Mead et al. (2013) showed large variability in DOM composition while considering the air-mass backward trajectories. Results of DOC concentrations in rainwater collected at environmentally different sites in central Poland reflected the influence of local, regional, and distant sources. The examples of air-mass trajectories from HYSPLIT simulations are presented in Fig. 5 so as to give an insight into the relationship between emission sources (regional/distant) and DOC concentrations in precipitation.

High DOC concentration (11.28 mg L^−1^) in the summertime rainwater sample from Poznań (02/07/13) was reported during the south to east advection when air masses arrived from Ukraine, Belarus, and southern Poland (Fig. 5(i)). For this rain episode, DOC was highly influenced by local/regional anthropogenic emission, i.e., high-temperature processes, industrial/urban activities, and traffic. In particular, fossil fuel combustion from Karolin power plants (located ca. 10 km to the northeast from the sampling site) was identified as a major anthropogenic contributor of carbonaceous particles (TC_rain_/Vw, *r* > 0.5). This enhancement of dissolved organic carbon in precipitation was also supported by wind, which had relatively low speed (0.4 to 2.7 m s^−1^) during those sampling days. It is well known that in urban areas, the anthropogenic emission is more influential on temporal variability of rainwater DOC than within suburban or rural sites (Sillanpää et al. 2012, Alvares et al. 2012). Although the input of various anthropogenic sources (i.e., transportation, industrial production) in Poznań is expected to be predominant, the role of biogenic emission in carbon budget cannot be negligible, especially during summertime.

In contrast, the highest value of DOC concentration (48.3 mg L^−1^) was observed for wintertime rain episode during western advection. The elevated DOC concentration in precipitation most likely reflected greater contribution of carbonaceous particles emitted from local/regional combustion activities (intensive fossil fuel use for house heating). However, as shown in Fig. 5ii, relatively low level of air mass travelling suggests that some fraction of total carbon measured in rainwater from Poznań could be transported from distant emission sources, such as highly industrialized areas in Germany, France, the UK, and Denmark.

The 3-day HYSPLIT simulation for the rain episode on 12 November 2013 showed that northern and northwestern winds prevailed. During this event, the DOC level was calculated to be 12.1 mg L^−1^ and was lower than the value measured in August (Fig. 5(i vs. iii)). The peak concentration of DOC in precipitation collected in November at this receptor site could be most probably related to regional anthropogenic sources (S to NW sector) due to the fact that Wielkopolski National Park has relatively low urban, industrial, and traffic emission compared to Poznań Agglomeration. However, it is also possible that a part of DOC measured in rainwater might originate from the outside of Wielkopolska Province. The good agreement of high rainwater DOC concentration with the HYSPLIT data occurs for relatively low level of air masses which were under both marine and continental influences. In addition, a significant contribution of polluted air masses transported over Baltic Sea region countries, Sweden, Lithuania, and Estonia, was observed (Fig. 5(iv)).

On the other hand, the high DOC concentration of 19.3 mg L^−1^ measured during the warm period in Jeziory could be most likely associated with biogenic emission (growing season, intense agricultural activities). These results are similar to other non-urban areas where higher DOC concentrations were strongly correlated with the emission from vegetation (Liu and Sheu 2003; Orlović-Leko et al. 2009). However, by analogy with the abovementioned examples, the impact of distant sources could also affect DOC levels in precipitation at Jeziory site. As illustrated in Fig. 5iii, the air parcel that passed over densely populated and industrialized regions in western Europe (UK, Netherland, Germany) and the North Sea could bring mixed fraction of organic particles towards the sampling site in Jeziory. Previous studies showed that when the level at which air masses travel slowly decreases with time, the time of residence of gaseous and particulate pollutants within the planetary boundary layer before arriving at the receptor site is longer (Siudek et al. 2015). Moreover, it was observed that higher concentrations of total organic carbon in aqueous phase (fog and cloud water) were associated with aged air masses (Ervens et al. 2013). The authors stated that a large fraction of organics transported in aged air masses is more highly oxidized and more soluble. The same mechanism can be important in relation to precipitation. For that reason, the impact of regional and distant sources on TC levels in rainfalls from central Poland cannot be negligible.

## Summary and conclusion

Concentrations of dissolved organic carbon, total carbon, and inorganic carbon were simultaneously determined in rainwater samples in the period between April and December 2013 at urban and forest sites in central Poland. It was found that the concentration range of DOC in urban precipitation was slightly higher than that at non-urban location. Moreover, the concentrations of dissolved organic carbon in rainwater from Poznań were higher as compared to other metropolitan cities in Europe, suggesting significant impact of local/regional anthropogenic sources. On the other hand, DOC levels measured in rainwater samples collected at Jeziory site were within the same range as literature values observed for other forested sites. The concentration of DOC in precipitation changed with seasons. For the whole sampling period, the seasonal differences of DOC concentration for the both selected sites were statistically significant. The statistically significant differences were proved by Kruskal-Wallis test (*p* < 0.05). During winter, DOC values were dominated by anthropogenic sources with a large contribution of local fossil fuel combustion processes (Poznań) and wood burning (Jeziory). In contrast, elevated DOC concentrations in rain samples, reported during warm season at the forest site, suggested significant contribution from biogenic emission. Based on backward trajectory analysis, it was demonstrated that high concentration value of DOC in rainwater at both sites coincided well with temporal evolution of air masses which passed over the industrially impacted regions in southern Poland and western Europe. Our preliminary results suggest that further investigations are needed to make a progress in understanding the role of removal processes and source-receptor relationships in local, regional, and global biogeochemical cycles of atmospheric carbon.
